# Serum Bilirubin as a Prognostic Factor in Lung Cancer

**DOI:** 10.3390/diagnostics16071001

**Published:** 2026-03-26

**Authors:** Antonios N. Gargalionis, Kostas A. Papavassiliou, Angeliki Margoni, Athanasios G. Papavassiliou

**Affiliations:** 1 Laboratory of Clinical Biochemistry, ‘Attikon’ University General Hospital, Medical School, National and Kapodistrian University of Athens, 12462 Athens, Greece; agargal@med.uoa.gr; 2 First University Department of Respiratory Medicine, ‘Sotiria’ Chest Hospital, Medical School, National and Kapodistrian University of Athens, 11527 Athens, Greece; konpapav@med.uoa.gr; 3 Department of Biological Chemistry, Medical School, National and Kapodistrian University of Athens, 11527 Athens, Greece; mmargoni@med.uoa.gr

Bilirubin, a metabolite of hemoglobin and a biomarker of liver function, has been considered for quite some time as harmful metabolic waste. Nevertheless, it has recently emerged as an endogenous, non-enzymatic antioxidant, scavenging peroxyl radicals and protecting against lipid peroxidation with potential antitumor effects [1]. Currently, there are independent studies regarding the role of bilirubin as an epidemiological, diagnostic, and prognostic biomarker in several types of solid tumors [1]. Although serum bilirubin seems to have a tumor-specific role, the general perception is that disease-free, slightly increased serum bilirubin levels are inversely correlated with cancer incidence and mortality, whereas excessive increase of bilirubin levels is associated with higher cancer incidence [1,2,3]. These observations are particularly evident in the case of lung cancer.

Different bilirubin levels have been detected in most studies between lung cancer cases and normal controls. An increase in baseline bilirubin serum levels has been inversely associated with lung cancer incidence in both sexes and all-cause mortality [4,5]. Low baseline levels of bilirubin (<0.75 mg/dL) have been specifically associated with a 55% increase in lung cancer incidence and a 66% increase in lung cancer mortality, compared to patients with higher baseline bilirubin levels (>1 mg/dL). In the same studies, every 0.1 mg/dL decrease in bilirubin was accompanied by a 5% increase in lung cancer incidence and 6% in lung cancer mortality in male smokers in particular [6]. With regard to the latter finding, smoking is a factor that further increases lung cancer risk independently in patients with low bilirubin [6]. It has long been established that smoking causes oxidative stress and inflammation with the production of reactive oxygen species (ROS), a known tumor facilitator [7]. Bilirubin scavenges ROS, decreases lipid peroxidation, and exerts an anti-inflammatory action. Therefore, lower serum bilirubin in high-risk patients potentially reflects inadequate protection for smokers.

Similar studies also reveal inverse association of bilirubin and lung cancer in men, among other antioxidant factors [8,9]. In a Japanese study with 403 lung cancer patients, the prediction model highlighted that lung cancer risk is associated with low bilirubin levels observed again in men, with the cut-off being <1.2 mg/dL. A mild increase in bilirubin levels, as well as a further increase in bilirubin levels (>1.2 mg/dL), are associated with a decreased risk of lung cancer in Japanese men among other types of solid cancers [10]. Corroborating findings in male Korean patients show that a 1-standard-deviation (SD) increase in total bilirubin is associated with an 18% lower lung cancer risk, and a 1-SD increase in indirect bilirubin levels is associated with an 11% decrease in lung cancer risk. This 1-SD increase in total bilirubin and direct bilirubin levels in the general population is correlated with a significant decrease in lung cancer risk in male smokers, whereas a 1-SD increase in total bilirubin is positively associated with a 15% higher lung cancer risk in women [11]. These data indicate that sex- and smoking-associated subgroups are relevant in the connection of bilirubin levels with the risk of lung cancer. Given the feasibility of testing, bilirubin could serve as a reliable biomarker for early screening, prognosis, and treatment-related decisions for these two subgroups.

Differences in baseline levels of bilirubin are also often the result of genetic modifications, and this seems to form a third subgroup in relation to lung cancer risk. United Kingdom (UK) data, including 377,294 participants, demonstrate a strong negative correlation of genetically raised bilirubin levels with lung cancer risk. Each 5 μmol/L increase of total bilirubin is associated with 1.2/10,000 person-years decrease in lung cancer incidence, which is further amplified for heavy smokers [12]. This appears to be the case with Japanese men mentioned earlier, who have elevated baseline bilirubin levels (>1.2 mg/dL) in conjunction with reduced lung cancer risk [10].

In addition to its role in risk stratification, several studies render bilirubin a prognostic and predictive biomarker in lung cancer. When total bilirubin, indirect bilirubin, and direct bilirubin before treatment were combined in one factor, patients with lower bilirubin levels had a shorter median progression-free survival than those with higher bilirubin levels (8 vs. 15 months), highlighting bilirubin as an independent prognostic factor [13]. In a separate study, Li and colleagues evaluated 1617 patients with normal bilirubin levels who had been subjected to complete resection for non-small cell lung cancer (NSCLC). Moderate presurgical high total bilirubin was linked with longer overall survival, disease-free survival, and distant metastasis-free survival. Similar results were reported with direct bilirubin and indirect bilirubin, the latter being highlighted as an independent prognostic factor [14]. Accordingly, prognostic data reinforces the protective role of higher bilirubin baseline levels in lung cancer. In terms of its role as a predictive biomarker, NSCLC patients with high direct bilirubin and smoking history exhibit shorter overall survival and higher mortality following therapy with epidermal growth factor receptor (EGFR)-tyrosine kinase inhibitor (TKI) [15]. Higher direct bilirubin is also associated with increased mortality in NSCLC patients with EGFR mutations [16]. Contradictory results in 131 lung adenocarcinoma patients, receiving EGFR-TKI, indicate that patients with elevated direct bilirubin present increased progression-free survival [17]. This discrepancy potentially reflects the dual role of bilirubin. On the one hand, high bilirubin may be associated with poor prognosis because of the complex interplay of EGFR signalling, hepatic metabolism, and oxidative stress in impaired liver function [18]. On the other hand, relation to increased progression-free survival establishes the beneficial antioxidant and protective role of bilirubin.

It is also important to assess the different roles of bilirubin as an indicator in the context of the albumin–bilirubin (ALBI) grade, a prognostic model including serum bilirubin and albumin levels that was described by Johnson and colleagues to evaluate liver dysfunction [19]. A meta-analysis involving 2057 patients showed that an increase in pretreatment ALBI grade is associated with poor overall survival and cancer-specific survival [20]. Similar results have been obtained for NSCLC patients and NSCLC patients treated with immune checkpoint inhibitors (ICIs) [21,22,23]. The platelet–albumin–bilirubin (PALBI) grade represents an additional adverse prognostic factor for overall and progression-free survival in small cell lung cancer (SCLC) [24]. With respect to high bilirubin levels within the ALBI index that are associated with worse survival, this observation may be attributed to lung cancer-associated liver metastases or impaired albumin concentration in patients with advanced cancer. Therefore, they should be explained in a different context than differences in baseline bilirubin levels. It seems that high bilirubin levels, in relation to low albumin, are linked to poor prognosis in established lung cancer, which reflects hepatic dysfunction, systemic inflammatory response, and malnutrition. This counteracts the protective role of mildly elevated bilirubin described in independent studies. Collectively, studies of bilirubin and bilirubin-based indices regarding clinical implications should be interpreted according to study design, especially between cohort and case–control studies. This is because they have different biases, generalizability, and effect measures that impact the strength of the findings.

There is currently limited mechanistic data concerning the role of bilirubin specifically in lung cancer cells. Bilirubin significantly reduces cell proliferation in human adenocarcinoma and arrests cell cycle at the G0/G1 phase, causing an increase in intracellular radical levels, DNA damage, and apoptosis and a decrease in mitochondrial function [25]. Exogenous administration of bilirubin via bilirubin nanoparticles (BRNPs) mediates tumor inhibition in NSCLC mice and promotes apoptosis of lung cancer cells and regulatory T (Treg) cell differentiation in vitro, simultaneously hampering the anti-apoptotic effect and T helper 17 (Th17) cell differentiation induced by the effect of cigarette smoke extracts. BRNPs upregulate the Treg/Th17 ratio and downregulate interleukin-6 (IL-6), interferon-γ (IFN-γ), and lipopolysaccharides (LPS), suggesting that bilirubin protects the patient from the progression of lung adenocarcinoma by repressing Th17 immune response and inflammation [26]. Conversely, inhibition of heme oxygenase-1 (HO-1), the enzyme that catalyzes the production of the bilirubin precursor termed biliverdin, suppresses A549 cell proliferation and migration [27]. Further experimental approaches are needed to clarify the mechanisms through which bilirubin achieves antitumor effects but also under which cellular status bilirubin presents tumor-promoting features.

In conclusion, large cohort studies render bilirubin’s role a promising field for further basic and clinical research in lung cancer. Consistent epidemiological findings showing inverse correlation with lung cancer risk, high feasibility of testing, and cost-effectiveness support bilirubin as a potential clinical biomarker for early detection, risk stratification, prognosis, and evaluation of therapeutic response in subpopulations, such as smokers. However, there are limitations that must be considered. Bilirubin-based laboratory methods demonstrate variability of sensitivity and specificity, whereas ALBI cut-offs are derived through receiver-operating characteristic (ROC) curve analyses and depend on the respective cohort, clinical subgroup, and disease parameters [15,17,20,28]. Furthermore, the potential of bilirubin as a screening strategy should not follow the one-size-fits all model, since this approach could increase overdiagnosis and false positive results, but instead emphasize specific population groups [29]. It should also be taken into account that bilirubin takes on a different role in the context of the ALBI grade, where elevated levels reflect hepatic impairment in established disease. Initial mechanistic data suggests that bilirubin uses immune cells of the tumor microenvironment (TME) to suppress tumor development. Future experimental investigations, in conjunction with well-characterized clinical cohorts, could determine whether bilirubin can be reliably incorporated as a prognostic and predictive biomarker in lung cancer.

## Data Availability

Not applicable.
